# Study design: two long-term observational studies of the biosimilar filgrastim Nivestim™ (Hospira filgrastim) in the treatment and prevention of chemotherapy-induced neutropenia

**DOI:** 10.1186/1471-2407-13-547

**Published:** 2013-11-16

**Authors:** Didier Kamioner, Stefan Fruehauf, Fréderic Maloisel, Laurent Cals, Stéphane Lepretre, Christian Berthou

**Affiliations:** 1 AFSOS and Hôpital Privé de l’Ouest Parisien, 78190 Trappes, France; 2 Paracelsus-Klinik, Center for Tumor Diagnostics and Therapy, Osnabrück, Germany; 3 Clinique Saint Anne, Department of Hematology and Oncology, Strasbourg, France; 4 CHRU de Besançon, Besançon, France; 5 Département d’Hématologie, Centre Henri Becquerel, Rouen, France; 6 Département d’Clinique Hématologie, Hôpital Morvan, Brest, France

**Keywords:** Granulocyte colony-stimulating factor, G-CSF, Filgrastim, Safety, Efficacy, Hospitalisation, Practice patterns, CD34, Chemotherapy, Neutropenia

## Abstract

**Background:**

Nivestim™ (filgrastim) is a follow-on biologic agent licensed in the EU for the treatment of neutropenia and febrile neutropenia induced by myelosuppressive chemotherapy. Nivestim™ has been studied in phase 2 and 3 clinical trials where its efficacy and safety was found to be similar to its reference product, Neupogen^®^. Follow-on biologics continue to be scrutinised for safety. We present a design for two observational phase IV studies that are evaluating the safety profile of Nivestim™ for the prevention and treatment of febrile neutropenia (FN) in patients treated with cytotoxic chemotherapy in general clinical practice.

**Methods/Design:**

The NEXT (Tolérance de Nivestim chez les patiEnts traités par une chimiothérapie anticancéreuse cytotoXique en praTique courante) and VENICE (VErträglichkeit von NIvestim unter zytotoxischer Chemotherapie in der Behandlung malinger Erkrankungen) trials are multicentre, prospective, longitudinal, observational studies evaluating the safety profile of Nivestim™ in 'real-world’ clinical practice. Inclusion criteria include patients undergoing cytotoxic chemotherapy for malignancy and receiving Nivestim as primary or secondary prophylaxis (NEXT and VENICE), or as treatment for ongoing FN (NEXT only). In accordance with European Union pharmacovigilance guidelines, the primary objective is to evaluate the safety of Nivestim™ by gathering data on adverse events in all system organ classes. Secondary objectives include obtaining information on patient characteristics, efficacy of Nivestim™ therapy (including chemotherapy dose intensity), patterns of use of Nivestim™, and physician knowledge regarding filgrastim prescription and the reasons for choosing Nivestim™. Data will be gathered at three visits: 1. At the initial inclusion visit, 2. At a 1-month follow-up visit, and 3. At the end of chemotherapy.

Recruitment for VENICE commenced in July 2011 and in November 2011 for NEXT. VENICE completed recruitment in July 2013 with 407 patients, and NEXT in September 2013 with 2123 patients. Last patient, last visit for each study will be December 2013 and March 2014 respectively.

**Discussion:**

The NEXT and VENICE studies will provide long-term safety, efficacy and practice pattern data in patients receiving Nivestim™ to support myelosuppressive chemotherapy in real world clinical practice. These data will improve our understanding of the performance of Nivestim™ in patients encountered in the general patient population.

**Trial registration:**

NEXT NCT01574235, VENICE NCT01627990

## Background

Chemotherapy-induced neutropenia (CIN) is a common and serious complication of myelosuppressive chemotherapy [1]. It is associated with significant morbidity and mortality [1], and can increase the overall cost of providing cancer therapy [2,3]. CIN can severely impair host defence systems, leading to an increased risk of life-threatening infections [3]. CIN may progress to febrile neutropenia (FN), which is an important contributor to chemotherapy-associated morbidity and mortality, and carries a high risk of death from systemic infection. Patients who develop FN (body temperature >38.5°C with absolute neutrophil count <0.5×10^9^/L) are stratified as high or low risk according to the Multinational Association for Supportive Care (MASCC) index. High-risk patients are generally treated in an in-patient setting with broad-spectrum intravenous antibiotics. Lower risk patients are also admitted to hospital, but can be managed with oral antibacterial agents [4].

FN can result in substantial dose reductions and delays to chemotherapy cycles that may jeopardise treatment outcomes, prolonging patient recovery, and diminish the success of antineoplastic therapy [5-7]. Dose reductions and delays in chemotherapy have been associated with worse patient outcomes in many cancers [5], including colorectal cancer [8], non-small-cell lung cancer [9] and breast cancer [10]. There is recent evidence that the impact on survival of chemotherapy dose modification may be lessened with newer therapies, such as platinum and taxane regimens in ovarian cancer [11] and cisplatin/carboplatin with vinorelbine/gemcitabine in NSCLC [12].

For over two decades, granulocyte colony-stimulating factors (G-CSFs; filgrastims) have been the mainstay of the treatment and prevention of CIN [13], where they have been found to reduce the risk of neutropenia across a range of patient settings, decrease the incidence of FN, reduce the incidence of infection, reduce the requirement for treatment with antibiotics, and accelerate neutrophil recovery [6,14-17]. Importantly, use of G-CSFs in patients undergoing myelosuppressive chemotherapy can reduce the need for physicians to delay chemotherapy cycles or reduce chemotherapy doses [18,19].

Along with the development of G-CSFs, advances in cancer care have been accompanied by reduced requirements for dose reduction and delay due to FN. A recent observational study of 1,849 patient records in the United States indicated that relatively few patients now receive high-risk regimens, and that treatment of FN to limit dose reduction and delay is chiefly reactive rather than prophylactic [20]. The apparent lack of prophylactic use of G-CSF may be driven in part by continuing debate over its survival benefits. A 2007 systematic review of 17 randomised controlled trials of G-SCF use in patients with solid tumours or lymphoma found that prophylactic G-CSF reduced the risk of FN and early death, including infection-related mortality [18]. In contrast, another meta-analysis conducted in the same year, this time on 148 trials, found no effect of prophylactic G-CSF on mortality, but did identify reduced risk of infection [21]. Elderly HER-positive breast cancer patients, specifically those receiving adjuvant dose-dense chemotherapy, also appear to derive no benefit from prophylactic G-CSF [22]. The most recent update of the EORTC guidelines on the use of G-CSF in chemotherapy patients acknowledges that data supporting prophylactic use are mixed, and echo the recommendations of the American Society of Clinical Oncology (ASCO) that prophylactic use of G-CSF should be reserved for high-risk (>20%) patients [6].

The cost of G-CSF use has come under scrutiny from various quarters, and ASCO has identified five key imperatives to improve cancer care at reduced cost, one of which is to ensure that prophylactic use of G-CSF is reserved only for patients with a high risk of FN (>20%), and where treatment regimens not requiring G-CSF are unavailable [23,24]. The approval of biosimilar versions of the originator products is increasing physician choice, improving patient access and has the potential to reduce costs associated with therapy [25]. Biosimilars are follow-on versions of peptide therapeutics and are produced by companies other than the company that marketed the original product. In contrast with small molecule generic drugs, it is not possible to make a completely identical copy of a peptide therapeutic, chiefly because, although the patent on the compound may have expired, the process used to make it remains proprietary, and various factors in the manufacturing and formulation processes can result in subtle variations between biosimilars and their reference product [26]. For these reasons, the European Medicines Agency (EMA) has produced several sets of stringent guidelines that must be adhered to in the development of biosimilar products [27-30]. These state that biosimilars must demonstrate similarity to the reference product in terms of molecular characterisation, purity, stability, pharmacokinetics, pharmacodynamics, clinical efficacy, tolerability and safety.

In the field of filgrastims, six biosimilar compounds have been approved according to the EMA standards, including Nivestim™ (Hospira filgrastim) [31]. Throughout the whole of its development programme, which has included a large-scale phase III clinical trial, Nivestim™, has demonstrated similarity in quality, safety and efficacy to its reference product Neupogen® [32-35]. However, despite proven similarity to Neupogen® in accordance with EMA standards, some physicians remain concerned over the safety of biosimilars in a range of indications, and seek reassurance from long-term safety studies [25,36-39].

With these concerns in mind, Hospira has designed two phase IV, post-marketing, longitudinal, observational, safety and efficacy studies that will gather additional long-term data to evaluate the safety profile of Nivestim™ for the prevention and treatment of FN in clinical practice given according to available clinical practice guidelines and the product label [6,40]. These studies are being conducted in France and Germany alongside an existing pan-European pharmacovigilance programme. In this paper we will describe the design of both of these studies.

## Methods

### Objectives

The authors present the design of two phase IV, longitudinal, observational, safety and efficacy studies that will evaluate the safety profile of biosimilar G-CSF (Nivestim™) for the prevention and treatment of FN in patients undergoing cytotoxic chemotherapy. The two observational studies are termed NEXT (Tolerance de Nivestim chez les patiEnts traités par une chimiothérapie anti-cancéreuse cytotoXique en praTique courante; NCT01574235) and VENICE (VErträglichkeit von NIvestim unter zytotoxischer Chemotherapie in der Behandlung maligner Erkrankungen; NCT01627990). The studies aim to evaluate the safety of a biosimilar G-CSF by producing evaluable long-term data on its use in a large patient population, without the constraints of a randomised controlled trial. The safety evaluation will include the recording of adverse events (AEs) in all system organ classes, as required by European Union (EU) pharmacovigilance guidelines.

In addition to safety parameters, the studies will also gather information on efficacy outcomes, patients’ characteristics, lab values, indications for treatment, patterns of use of Nivestim™, evaluation of dose intensity, physician knowledge regarding G-CSF prescription and the reasons for choosing Nivestim™ over other available therapies. The VENICE study will also collect data on levels of CD34+ cells in the blood, as this may have predictive value as a marker of stem-cell reserves and regenerative capability [41-43].

### Study populations

VENICE is currently being conducted in Germany and NEXT is being conducted in France – both studies are being conducted by practicing oncologists and haematologists. VENICE and NEXT began recruitment in July 2011 and November 2011, respectively, and are recruiting subjects from 70 sites in Germany and 160 sites in France. Site selection is based on their particular focus on oncology and haematology. NEXT aimed to recruit 2,000 adult patients in total, while VENICE aimed to recruit 700 adult and paediatric patients (no age limits). Both studies will recruit over a period of 24 months with a maximum follow-up period of 6 months for each patient.

The studies will include three patient visits: 1. Inclusion visit. 2. First follow-up visit after the first course of Nivestim™. 3. Second follow-up visit at the end of the chemotherapy regimen after the last cycle of chemotherapy. The patient information collected at each visit is shown in Table 1.

**Table 1:** Information included on case report forms for baseline and follow-up visits

**Baseline visit**	**Follow-up visits**
• Informed consent	• ANC-nadir, ANC-value
• Criteria for inclusion and exclusion	• AEs (hospitalisation, clinically relevant)
• General information relating to the patient being	• Vital parameters
• treated (status, demographic)	• Blood tests
• Vital parameters	• Description of chemotherapy including dose modifications
• Medical and surgical anamnesis (FN, recurrent infections, HIV, COPD, cardiovascular diseases, renal or hepatic insufficiency)	• Prescription of Nivestim™
• Preceding treatments (chemotherapy, radiotherapy and/or surgery)	• Withdrawal before end of study, including reasons and date
• Information relating to pathology of malignant haemopathies (Hodgkin’s, non-Hodgkin’s, TNM, stem-cell transformations)	• Concomitant therapies.
• Information relating to the pathology of solid tumours (localisation, TNM)	
• Laboratory (haemoglobin, thrombocytes, leukocytes, neutrophil, CRP, CD34+ cell count)	
• Infections (type, localisation, additional factors [hypotension, dermatitis, erysipelas, sepsis])	
• Description of chemotherapy (aims, schedule, duration, cycles, active principle)	
• Prescription of Nivestim (primary/secondary prophylaxis, dosage, duration, antibiotic, time-frame.	

*Abbreviations*: *ANC* absolute neutrophil count, *HIV* human immunodeficiency virus, *COPD* chronic obstructive pulmonary disease, *CRP* C-reactive protein, *AEs* adverse events.

Inclusion and exclusion criteria are shown in Table 2.

**Table 2:** Inclusion and exclusion criteria for NEXT and VENICE

**Inclusion criteria**	**Exclusion criteria**
• Adult patients (NEXT)	• Chronic myeloid leukaemia
• Adult and paediatric patients (VENICE)	• Myelodysplastic syndrome
• Solid tumours or haematological malignancies	• Hypersensitivity to the active substance
• Treated or planned treatment with cytotoxic chemotherapy irrespective of cycle	• Hypersensitivity to one of the excipients of Nivestim™
• Indicated for G-CSF therapy according to the product label for Nivestim™	• Undergoing treatment with G-CSF for ongoing FN (curative) (VENICE only)*

*NEXT includes two patient profiles: curatively and prophylactically treated patients; VENICE includes only patients treated prophylactically. Patients are not excluded if they have congenital neutropenia disorders or have received stem cell transplants.

### Treatment interventions

In clinical practice, primary prophylaxis of CIN with a filgrastim is recommended in patients undergoing chemotherapy where the estimated risk of developing FN is ≥20%, or for patients undergoing chemotherapy with FN risk factors and a risk of developing FN of ≥10%. Secondary prophylaxis with filgrastim is recommended only for patients in whom a dose reduction would compromise therapy success or result in a reduction of overall survival, or in patients who have experienced FN with previous treatment. In line with these recommendations, the NEXT and VENICE studies, will allow the use of Nivestim™ for both primary and secondary prophylaxis. The prophylactic dosage will be s.c. or i.v. Nivestim™ 5 μg/kg/day, and the first injection will be administered between 24 and 72 hours after the cytotoxic chemotherapy. G-CSF therapy will continue until the expected time the neutrophil nadir has passed or until the post-nadir blood neutrophil count returns to a level within the normal range (above 1,000 cells/mm^3^).

Curative treatment (treatment for ongoing FN) is also possible in NEXT and VENICE; however, patients treated in this manner will be omitted from the documentation and analysis. The curative use of G-CSF will be limited to patients with FN and patients with signs of serious infection such as major tissue or fungal infection, and the dose used will be the same as that for prophylactic therapy. In curative circumstances, and in common with prophylactic use, G-CSF therapy will continue until the post-nadir blood neutrophil count is above 1,000 cells/mm^3^. Studies of filgrastim in patients with severe impairment of renal or hepatic function have shown it exhibits similar pharmacokinetic and pharmacodynamic profiles to those seen in normal individuals; therefore, it is not necessary to modify the Nivestim™ dose in patients with renal or hepatic dysfunction [40].

### Endpoints

NEXT and VENICE are both gathering similar endpoints on the safety and clinical efficacy of Nivestim. The primary endpoint is the safety and tolerability of Nivestim™ evaluated by the collection of data on all AEs experienced by patients as well as the incidence of and reasons for hospitalisation. The secondary endpoints are varied and include efficacy and practice pattern data. For baseline patient characteristics, the study will record data on socio-demographics, surgical and therapeutic medical histories, the nature of the patients’ malignancies (including location and stage), details of previous chemotherapy received, and clinical and laboratory parameters before treatment with Nivestim commenced. In the VENICE study only, data will be recorded on the CD34+ count at baseline. In the analysis, the CD34+ count will also be used to stratify patients according to high (i.e. good haematopoeitic reserve) and low (poor haematopoetic reserve) values at Visit 1. In studies of patients with Hodgkin’s and non-Hodgkin’s lymphoma, CD34+ levels can predict the progenitor cell yields [41,42].

For the efficacy endpoints, both studies will assess the clinical effectiveness of Nivestim™ according to the duration of neutropenia (number of days with grade 4 neutropenia with an ANC <0.5×10^9^/L), the incidence and duration of FN (body temperature of >38.5°C for more than 1 hour and ANC <0.5×10^9^/L on the same day), the occurrence of infection, delay of chemotherapy cycles, chemotherapy dose reductions due to neutropenia, and circulating ANC levels on the final day of filgrastim therapy.

Using a questionnaire, the studies will also collect data concerning the mode of treatment of patients with Nivestim™, including indication for treatment, dose, route of administration, treatment duration, and any delay in the initiation of Nivestim™ treatment owing to chemotherapy treatment. Timings of ANC nadir and chemotherapy adjustments will also be recorded.

To understand the factors that influence prescribing behaviour for biosimilar filgrastims, both studies will collect data on physician age, sex, specialty, practice structure and job title. Additionally, their general and specific G-CSF prescribing habits will be observed.

### Statistical analysis

NEXT and VENICE will analyse the outcomes for all subjects and all researched study populations – all data will be described in the analyses and missing data will not be substituted. The analysis sets for the studies will comprise an all-treated set, a safety set, an efficacy set and an investigator set.

The all-treated set will be made up of patients who have received Nivestim™ at least once, and whose enrolment visit was documented. The safety set will include patients from the all-treated set for whom at least one further visit has been documented. A sub population analysis of the safety set will involve stratification by age (<18, 18–65, >65 years), and stratification by tumour type (haematological versus solid).

The efficacy set will include patients from the safety analysis set who fulfil all the inclusion and exclusion criteria and whose baseline ANC value and one other ANC value (ANC-nadir/ANC-value) has been documented either during the therapy or shortly afterwards. In VENICE, patients from the efficacy analysis set will be stratified according to baseline CD34+ cell counts (low or high).

The investigator set will include all participating physicians who have at least partially completed the Physician’s Questionnaire.

Endpoints with quantitative variables will be described in terms of the number of values, the number of missing values, means, medians, standard deviation, minima and maxima. Endpoints with qualitative data will be represented with percentages and frequencies. The duration of each patient’s participation in the studies will be factored such that incidence rates can be extrapolated to patient-years.

The level of significance for the two observational studies has been set at 0.05; however, NEXT and VENICE have used different methods for estimating the required sample size to accurately represent the proportion of patients who will be hospitalised because of FN or infection during the study. For the NEXT study, the sample size was based on the results of a 2003 oncology survey conducted by Louis Harris & Associates of 285,000 cancer patients treated with cytotoxic chemotherapy. Of these patients, 68,000 received G-CSF treatment. Using this figure of 68,000 patients, enrolment of 2,000 patients is required for NEXT to identify events in the overall population that are predicted to occur at an incidence of 1 ± 2.16%. For VENICE, the investigators calculated the required sample size by using an estimation of the proportion of patients who will be hospitalised due to FN or an infection during the observational phase of the study. In this case, assuming a maximum probability for the occurrence of hospitalisation due to FN or infection is 20% for each patient recruited, a sample size of n = 683 patients is needed to attain a 95% confidence interval (CI) with an accuracy level of ±3%. Taking into account a potential drop-out rate of 2.5%, 700 patients are required for VENICE.

For the safety analysis, the average time of occurrence of each type of AE will be calculated, and results will be described using descriptive statistics. Additionally, the characteristics of patients with each type of AE will be described in detail. The categorisation for the evaluation of AEs is given in Table 3.

**Table 3:** Evaluation of AEs in NEXT and VENICE

All AEs will be evaluated according to:	
•	Clinical relevance
•	Degree of seriousness (if deemed serious, a description of the outcome of each AE, e.g. death, life-threatening, hospitalisation, will be given)
•	Intensity (mild, moderate, severe)
•	Outcome (restoration, persistent damage, etc.)
•	Relationship to G-CSF therapy (none, possible, probable)
•	Occurrence of AEs relative to the first administration of Nivestim treatment
•	Any measures taken with regard to Nivestim™ therapy (none, reduction of dose, discontinuation, temporary suspension).

During both studies, the number of patients requiring hospitalisation will be recorded together with the reasons for hospitalisation. The incidence of hospitalisation due to FN or infection will be represented as absolute and relative frequencies (%, patient-years). Potential risk factors for the occurrence of hospitalisation will be identified and quantified using a multivariate survival analysis (Cox regression). Cases of hospitalisation will be described in detail, including the reason (FN or infection [bacterial, fungal or viral]), the duration of FN or infection, and the start time of FN or infection relative to the administration of Nivestim™.

All statistical analyses will be performed using SAS version 9.13 or later.

### Study limitations

Clinical trial populations are a narrow representation of the population of patients encountered in routine clinical practice. Both the NEXT and VENICE studies present an opportunity to record and analyse the performance of a biosimilar filgrastim over an extended period in a diverse group of patients. However, the study designs have some inherent limitations. For NEXT, the diversity of the patient characteristics may create statistical noise in the analyses, but this may be lessened by the large sample size. Additionally, due to the observational nature of the NEXT study there is no control arm to enable comparison of the performance of different filgrastims in the treatment of CIN. VENICE shares the general limitations of NEXT, but also has a relatively low sample size, and includes a mixture of adults and paediatric patients that may confound analyses.

## Discussion

**Figure 1:**
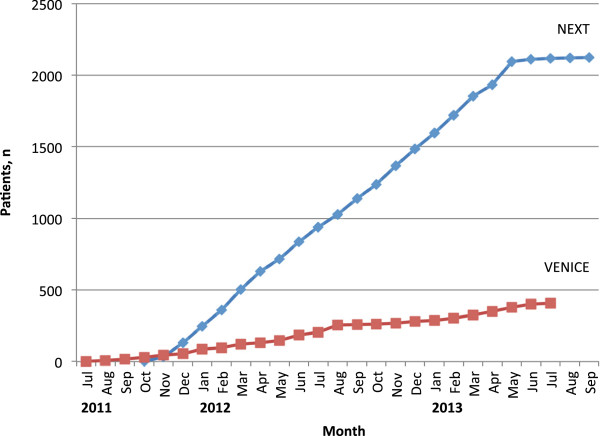
Enrolment into NEXT and VENICE as of 31 September 2013.

Recruitment began in July 2011 for the VENICE study and in November 2011 for the NEXT study. As of September 2013, both studies have completed recruitment. VENICE has 407 patients enrolled and NEXT has 2123 patients (Figure 1). Currently, both studies are still on course to conclude as planned – VENICE will be completed in December 2013 and NEXT will be completed in March 2014.

The NEXT and VENICE studies will provide additional information on the long-term safety and efficacy of Nivestim™ in patients being treated for malignancy with cytotoxic chemotherapy. The growing prominence of biosimilars among the therapeutic armamentarium and the improved access that they offer means that confidence in their safety is paramount.

In accordance with the standards set by the EMA [27-30], studies on Nivestim™ have demonstrated that it is equally as effective as the reference product, Neupogen®, and has a similar safety profile [33-35]. A phase III randomised equivalence trial in 279 breast cancer patients found that the mean duration of severe neutropenia (DSN) was 1.3 days for Neupogen® and 1.6 days for Nivestim™, which met the protocol-defined criteria for bioequivalence [35]. Secondary endpoints also showed Nivestim™ to be equivalent to Neupogen® in terms of time to absolute neutrophil count (ANC) recovery and incidence of FN [35]. These data supported the findings of two phase I trials studying the pharmacokinetics and pharmacodynamics of Nivestim™. In these studies, the efficacy, safety and pharmacokinetic characteristics of Nivestim™ and Neupogen® were found to be equivalent in healthy volunteers [33,34]. The studies performed with Nivestim™ were sufficient to satisfy the EMA that the quality of the drug and its performance versus the reference compound Neupogen® was sufficient to warrant approval for the treatment and prevention of neutropenia in patients undergoing myelosuppressive chemotherapy and those with severe idiopathic neutropenia, those with neutropenia arising from primary HIV infection and for the mobilisation of peripheral blood stem cells [40].

As more biosimilar filgrastims have become available, the pharmaceutical industry and the clinical community have continued to watch with interest how this relatively new class of agents performs in patients and whether any differences in the agents may become apparent as time passes and increasing numbers of patients are treated. The biosimilar manufacturers have continued to support investigation of their products even after they have been approved, and differences in performance between biosimilar drugs and the reference product have been found to favour biosimilars. For instance, studies of the biosimilar filgrastim XM02 have found the level of impurities evident in the XM02 product to be less than those found in Neupogen® [36]. Additionally, the thermal stability of Nivestim™ has been shown to be sufficient to enable out-of-fridge storage for up to 7 days without appearance of impurities or additional degradation products – a property not shared with other filgrastims [44].

NEXT and VENICE come at a time regulators are seeking data from long-term observational studies in clinical practice. In many cases, regulatory agencies will approve a drug for clinical use and ask that additional data be gathered via pharmacovigilance programmes or via specifically designed phase IV clinical trials. In the UK, the Medicines and Healthcare products Regulatory Agency MHRA has been examining its approval process for medicines, and has gone as far as to suggest that certain new agents should receive an accelerated provisional approval based on registrational clinical trials, and that drugs should then be subject to close monitoring in a 'real world’ setting prior to full approval [45]. The same agency has contributed to the UK Clinical Research Collaboration report and the National Institute for Health Research’s Research Capability programme, which together have demonstrated the possibility for collecting long-term data on a large scale [46,47]. The MHRA has concluded that population-based data will have a beneficial effect on observational and interventional research, and the agency has also suggested that it is prepared to assist in the development of surveillance methodologies to achieve this [48]. It is therefore important to ensure that physicians and regulatory agencies have access to comprehensive data that accurately reflects the performance of biosimilar filgrastims in the clinic.

## Conclusion

Owing to the duration of the studies, the large number of patients planned for enrolment in multiple centres, and the clinical practice setting of NEXT and VENICE, these studies will produce a significant amount of comprehensive, high-quality, evaluable data relating to the safety, efficacy and clinical use of Nivestim™. The large cohort of patients, particularly for NEXT, will enable the objective evaluation of the safety and efficacy profiles of Nivestim™.

Access to filgrastims remains limited for many patients and physicians in the EU [25]. The NEXT and VENICE studies will provide healthcare agencies with much needed additional data, and may assist in maintaining and expanding access to biosimilar filgrastims for the treatment of CIN.

Observational studies of differing designs have been conducted in chemotherapy-induced FN in the past. A 2009 retrospective survey of patient cases in the United States examined the prevalence of FN and the practices associated with the delivery of filgrastim and pegfilgrastim. Pegfilgrastim was associated with a lower incidence of FN than filgrastim, which was not evident in the clinical trials for pegfilgrastim [49]. This finding underscores the need for observational studies to reveal differences that do not emerge in clinical trials, and highlights that variations in real world clinical practice can have an impact on the overall effectiveness of therapeutic interventions.

The value of observational studies has been further highlighted by an observational study that examined G-CSF use in chemotherapy principally from the point of view of cost [20]. This study of 1,849 lung and colorectal cancer patients found that 17% and 18% of patients on high-risk and intermediate-risk chemotherapy regimens received G-CSF, in contrast to 10% on low-risk regimens. Patients enrolled in a health maintenance organisation (HMO) were less likely to receive G-CSFs compared with non-HMO patients [20]. Neither of these findings are particularly surprising; however, the study also found that 96% of patients were receiving G-CSF in clinical situations outside of label- and guidelines-mandated indications [20]. This is a major deviation from recommended practice, the type of which could only be revealed in an observational study. These kinds of data support the notion of ASCO that there are opportunities to improve care and reduce the cost of G-CSF therapy [23]. NEXT and VENICE are designed differently, and use label-mandated posology for the administration of G-CSF. In this way, the NEXT and VENICE trials will answer different clinical questions to existing observational studies, and thereby add to the totality of real world data on supportive care for cancer.

## Competing interests

All authors have received funding from Hospira for the conduct of the studies in their institutions.

## Authors’ contributions

All of the authors were involved in the design of the study and all are involved in the collection, analysis and interpretation of data from enrolled patients in their institution. All authors read and approved the final manuscript.

## Pre-publication history

The pre-publication history for this paper can be accessed here:

http://www.biomedcentral.com/1471-2407/13/547/prepub
